# The Editor's Letter-Box

**Published:** 1915-06-19

**Authors:** 


### THE EDITOR'S LETTER-BOX.

### War Service Badges for Hospital Staffs.

*To the Editor of* The Hospital.

"Hospital Secretary."

*To the Editor of* The Hospital.

Sir,—Hospital and institutional officers generally will welcome your action in dealing with the question of badges.

As an infirmary official (married) and of Army age, I have been repeatedly interrogated by recruiting sergeants as to enlisting. The question is one for each individual's conscience, and, whilst I am quite willing to enlist, my own decision is that I am serving the country more usefully by plodding away at my work—the nature of which is skilled, and for which a substitute is difficult to obtain.

Whilst the larger hospitals can no doubt issue a suitable badge, the matter is more difficult in institutions where perhaps only two or three of the male staff are of Army age.

Could not badges be issued to each medical superintendent for members of his staff who are, in his opinion, performing useful and necessary duties?—Yours faithfully,

"Semi-Professional."

June 12, 1915.

[On page 260 of our present issue will be found an illustration and an article dealing with the points raised by "Hospital Secretary" and "Semi-Professional."—Ed. The Hospital.]

